# Clinical and financial outcomes of hospitalizations for cardiac device infection during the COVID-19 pandemic in the US

**DOI:** 10.1371/journal.pone.0291774

**Published:** 2023-09-20

**Authors:** Nameer Ascandar, Nikhil Chervu, Syed Shahyan Bakhtiyar, Nam Yong Cho, Shineui Kim, Manuel Orellana, Peyman Benharash

**Affiliations:** 1 Cardiovascular Outcomes Research Laboratories (CORELab), David Geffen School of Medicine, University of California, Los Angeles, California, United States of America; 2 Depatment of Surgery, David Geffen School of Medicine, University of California, Los Angeles, UCLA, Los Angeles, California, United States of America; 3 Department of Surgery, University of Colorado, Aurora, Colorado, United States of America; 4 Division of Cardiac Surgery, David Geffen School of Medicine, University of California, Los Angeles, UCLA, Los Angeles, California, United States of America; Juntendo University: Juntendo Daigaku, JAPAN

## Abstract

**Background:**

Cardiac device infection (CDI) can occur in up to 2.2% of patients after device placement, with mortality rates exceeding 15%. Although device removal is standard management, the COVID-19 pandemic has been associated with resource diversion and decreased patient presentation for cardiovascular disease. We ascertained the association of the COVID-19 pandemic with outcomes and resource utilization after admission for CDI.

**Methods:**

The 2016–2020 National Inpatient Sample was used to retrospectively study all adult admissions for CDI. Patients admitted between March and December, 2020 were classified as the pandemic cohort, with the rest pre-pandemic. The primary outcome was major adverse events (MAE), with secondary outcomes of overall length of stay (LOS), post-device removal LOS, time to device replacement, and hospitalization costs. MAE was a combination of in-hospital mortality and select complications. Multivariable regression models were developed to determine the relationship between the pandemic and the aforementioned outcomes.

**Results:**

Of an estimated 190,160 patients, 14.3% comprised the pandemic cohort; 2.4% of these patients were COVID-19 positive. The pandemic cohort was older, less commonly female, and had higher rates of congestive heart failure. After adjustment, the pandemic was not associated with altered odds of MAE, device removal, or subsequent device replacement. The pandemic was, however, associated with decreased adjusted overall LOS (β -0.38 days) and days to device replacement (β -0.83 days). The pandemic was likewise associated with $2,000 increased adjusted hospitalization costs.

**Conclusion:**

The pandemic did not have a significant impact on clinical outcomes in patients admitted for CDI, despite higher hospitalization costs and decreased length of stay.

## Introduction

With incremental technologic advances and the availability of evidence-based guidelines, the implantation of intra-cardiac devices has seen a substantial rise in recent years [1]. Permanent pacemakers and defibrillators are increasingly implanted in patients for cardiac resynchronization therapy and prevention of sudden death [2]. However, up to 2.2% of patients experience cardiac device infections (CDI) with reported mortality as high as 16.9% [3,4]. Furthermore, such infections are associated with substantial morbidity and resource use. This is, in part, attributable to the need for total removal of implanted device followed by reimplantation in select cases [5–7].

The coronavirus disease 2019 (COVID-19) pandemic stained the US healthcare system at unprecedented levels. In particular, public anxiety and resource diversion may have significantly impacted the management of cardiovascular disease [8,9]. While hospitalizations due to critical respiratory illness surged, several investigators have reported a significant decline in admissions for urgent cardiovascular diseases [10]. Wu and colleagues reported a 42.3% decline in hospitalization for acute myocardial infarction, but higher 30-day mortality in those presenting within the first 2 months of the pandemic in the United Kingdom [8]. Furthermore, a multicenter study by Boriani and colleagues reported a greater than 50% reduction in the number of implantations of cardiac electronic devices during the pandemic [11]. However, trends and outcomes of CDI during the pandemic have not been evaluated thus far.

The present study examined trends of CDI incidence and the association of the COVID-19 pandemic period with clinical and financial outcomes in a national cohort of patients hospitalized with CDI. We hypothesized the COVID-19 pandemic to be associated with increased mortality, complications, as well as greater hospitalization costs compared to the pre-pandemic period.

## Materials and methods

### Data source and study population

**Fig 1:**
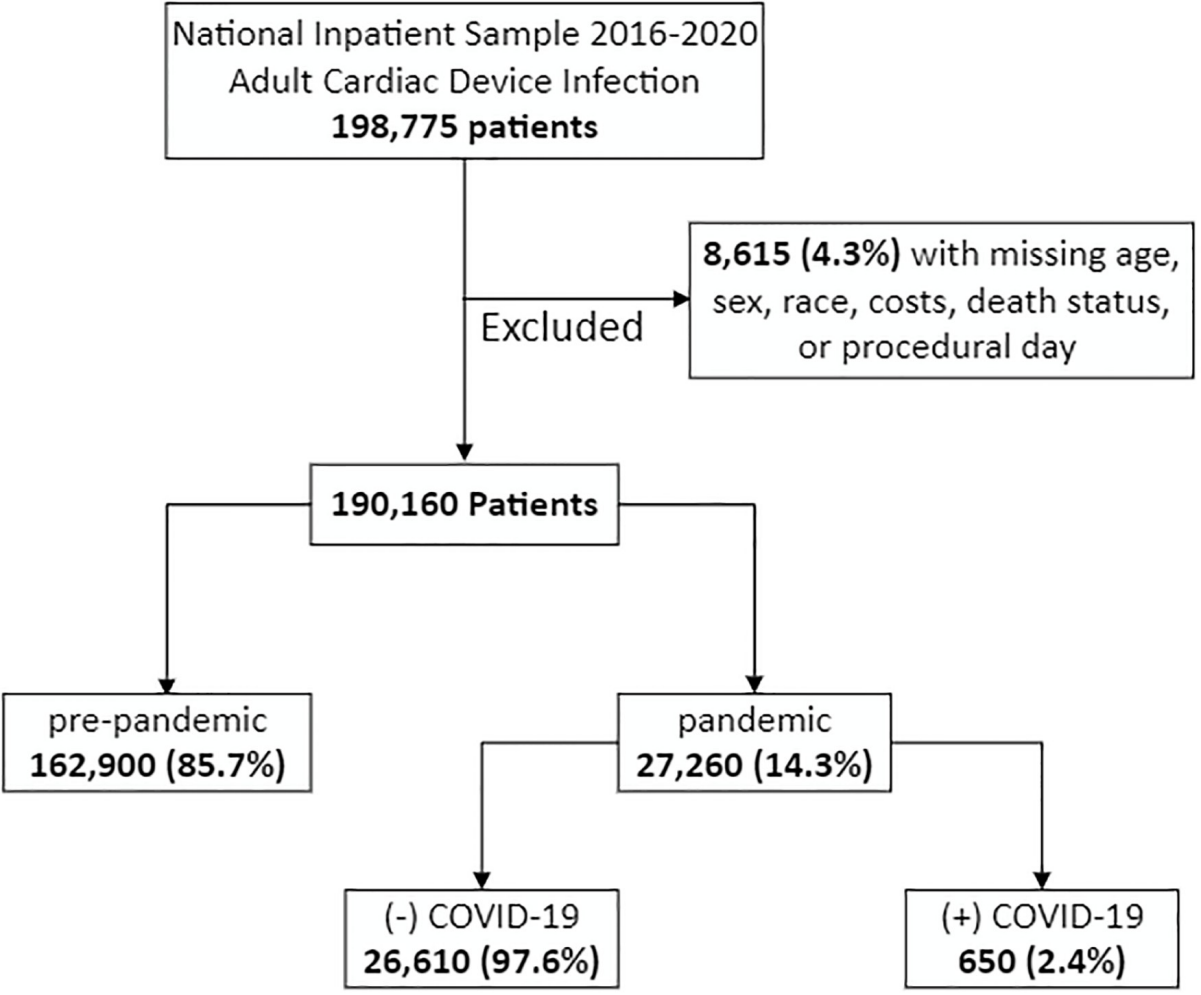
Exclusion criteria.

This was a retrospective study done in 2023 using the 2016–2020 National Inpatient Sample (NIS). Using *International Classification of Diseases*, *Tenth Edition* (ICD-10) code T82.7XXA, we identified all adult (≥18 years) admissions with a primary diagnosis of CDI. Maintained by the Agency for Healthcare Research and Quality as part of the Healthcare Cost and Utilization Project (HCUP), the NIS is the largest publicly available all-payer inpatient database providing accurate estimates for approximately 97% of annual US hospitalizations [12]. Patients with missing data for age, sex, race, costs, death status, or procedural day were excluded from the analysis (4.3%; Fig 1). Accounting for sampling differences and clustering, HCUP provides trend and discharge weights to generate national estimates of all inpatient hospitalizations. All analyses used survey-weighting methodology to estimate a national sample. The NIS accrues inpatient data from 48 states and includes information regarding patient demographics, hospital characteristics, as well as diagnoses and procedures using ICD-10 codes.

### Variable definitions and study outcomes

Patient and hospital characteristics were defined using the HCUP data dictionary [12]. Additional characteristics were tabulated using ICD-10 codes. Patients admitted from March through December of 2020 were stratified into the pandemic cohort, with the rest comprising the pre-pandemic cohort. The van Walraven modification of the Elixhauser Comorbidity Index, a previously validated composite score using 30 chronic conditions, was used to quantify the burden of chronic disease [13]. Hospitalization costs were derived by applying hospital-specific cost-to-charge ratios to total charges with inflation-adjusted to the 2020 Personal Healthcare Index [14]. The primary outcome of interest was in-hospital major adverse events (MAE), while secondary outcomes were overall duration of hospital stay (LOS), post-device removal LOS, time to device replacement, and hospitalization costs. MAE was defined as a composite of in-hospital mortality, stroke/transient ischemia attack, respiratory complications (acute respiratory distress syndrome, acute respiratory failure, prolonged ventilation, pneumothorax, pneumonia and empyema), cardiac arrest, gastrointestinal bleeding, acute kidney injury requiring dialysis, thromboembolic complications (deep venous thrombosis or pulmonary embolism), wound dehiscence, sepsis, and SIRS with end-organ dysfunction.

### Statistical analysis

Categorical and continuous variables are reported as frequencies (%) and mean with standard deviation (SD), respectively. Continuous variables that demonstrated skewed distribution were reported as medians and interquartile ranges (IQR). Pearson’s chi-squared and adjusted Wald test were used to analyze the significance of intergroup differences for categorical and continuous variables, respectively. Prior to regression, relevant patient and hospital characteristics were selected for analysis via the Least Absolute Shrinkage Selection Operator (LASSO). This regularization method that reduces variable collinearity while improving the accuracy and out-of-sample reliability of prediction models [15]. Multivariable linear and logistic regression models were developed to evaluate the association between time to procedure and the above outcomes of interest. Models were evaluated using receiver-operating characteristics (C-statistic) as well as Akaike and Bayesian information criteria, as appropriate [16]. Regression outcomes are reported as adjusted odds ratio (AOR) for dichotomous variables and beta coefficients (β) for continuous variables with 95% confidence intervals (95%CI). All statistical analyses were performed using Stata 16 (StataCorp, College Station, TX), with an α less than 0.05 set for statistical significance. The Institutional Review Board at the University of California, Los Angeles, deemed this study exempt from full review.

## Results

### Demographics and clinical characteristics

Of an estimated 190,160 patients who met inclusion criteria, 27,260 (14.3%) comprised the pandemic cohort. Among the patients in this group, 650 (2.4%) were noted to be COVID-19 positive. Compared to pre-pandemic, the pandemic cohort was older (62.3 ± 15.3 vs 61.8 ± 15.5 years, P = 0.02), less commonly female (38.9 vs 42.2%, P<0.001), and had a higher mean Elixhauser Index (5.5 ± 2.1 vs 5.3 ± 2.1, P<0.001). In addition, pandemic patients had higher rates of congestive heart failure (47.3 vs 38.7%, P<0.001), and peripheral vascular disease (25.4 vs 21.8%, P<0.001). Both cohorts were similar in racial composition (Table 1).

**Table 1:** Baseline patient, clinical, and hospital factors after admission for cardiac device infection during and prior to the COVID-19 pandemic.

	pandemic (n = 27,260)	pre-pandemic (n = 162,900)	P-value
Age (years, mean ± SD^†^)	62.3 ± 15.3	61.8 ± 15.5	0.02
Female (%)	38.9	42.2	<0.001
Elixhauser Index (mean ± SD^†^)	5.5 ± 2.1	5.3 ± 2.1	<0.001
Device Replacement (%)	4.7	4.7	0.83
Device Removal (%)	17.3	15.3	0.004
Race (%)			0.51
White	55.3	53.8	
Black	30.0	28.3	
Hispanic	10.6	11.4	
Asian/Pacific Islander/Other	6.1	6.5	
Hospital status (%)			<0.001
Urban Teaching	82.6	78.5	
Urban Non-Teaching	13.3	17.6	
Rural	4.1	3.9	
Comorbidities (%)			
Bacteremia	13.1	11.1	<0.001
Coagulopathy	16.6	15.7	0.08
Congestive Heart Failure	47.3	38.7	<0.001
Diabetes	48.3	49.6	0.12
Endocarditis	12.9	11.0	<0.001
Hypertension	85.9	85.3	0.32
Liver Disease	6.7	6.7	0.97
Metastatic Cancer	1.7	1.8	0.63
Neurological Disorders	17.4	16.0	0.01
Obesity	22.6	20.5	<0.001
Peripheral Vascular Disease	25.4	21.8	<0.001
Pulmonary Circulation Disorders	9.2	9.5	0.55
Renal Failure	64.6	66.9	0.01
Rheumatoid Arthritis	4.5	4.3	0.49
Tumor	3.4	3.4	0.94

^†^ SD, *Standard Deviation*.

### Trends in overall hospitalization for CDI and device removal

**Fig 2:**
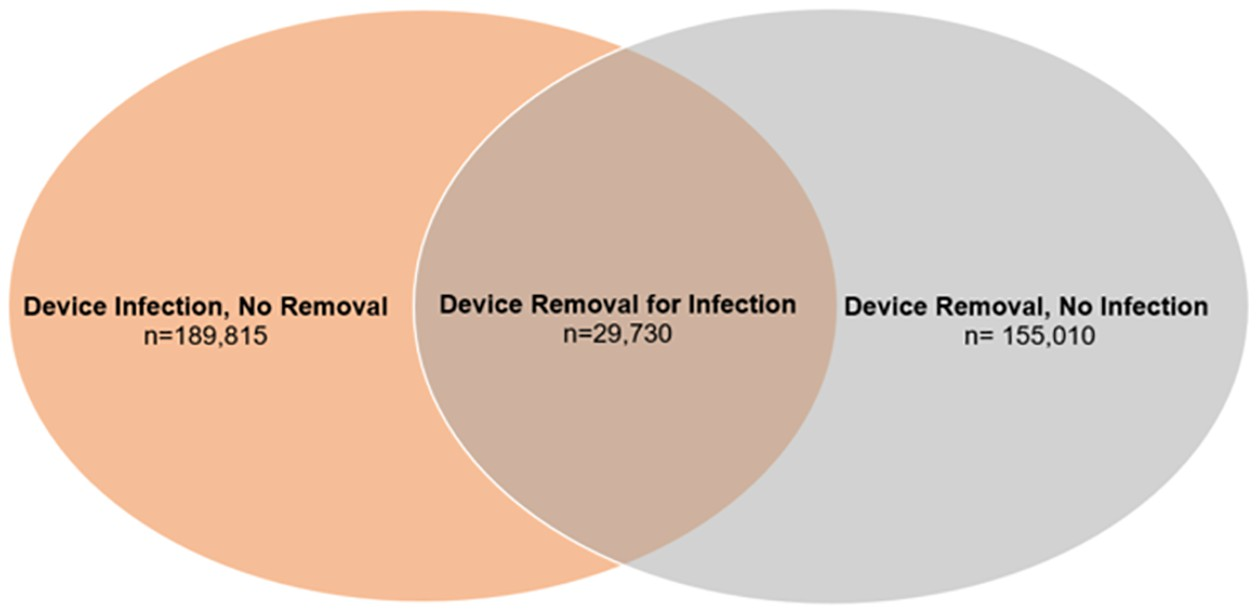
Venn diagram illustrating volume distribution of device removal.

**Fig 3:**
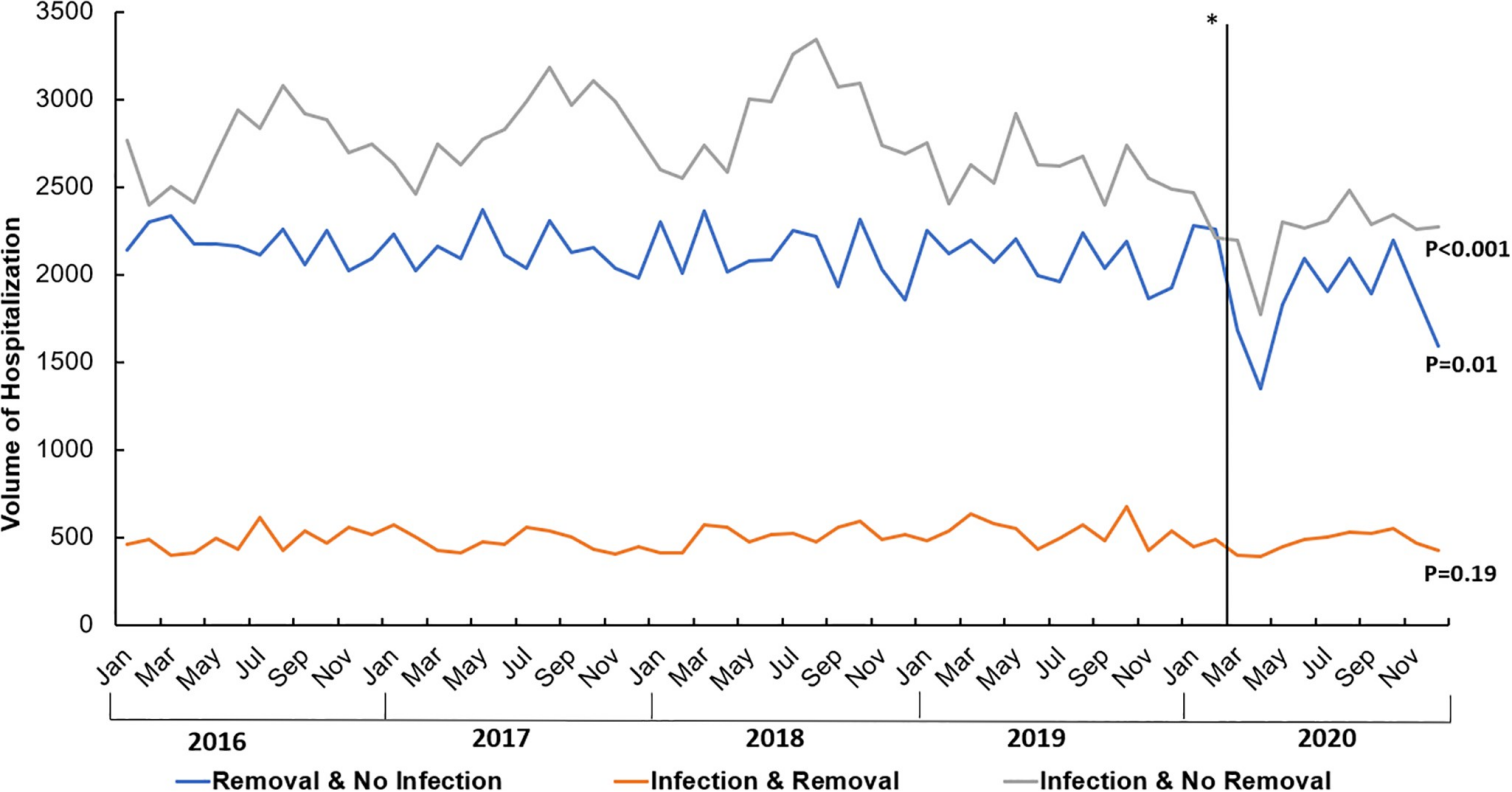
Trends in the monthly volume of cardiac device infection and removal. *Solid line—beginning of pandemic.

On a separate time trend analysis, we identified all adult hospitalizations entailing either device removal or device infection (Fig 2). The mean number of total device removal decreased during the pandemic (2,324 ± 306 vs 2,635 ± 147, p = 0.01) compared to before. Similarly, there was a decrease in the mean number of total CDI hospitalizations during the pandemic (2,721 ± 225 vs 3,252 ± 261, p<0.001) compared to pre-pandemic. The monthly volume of CDI patients admitted for device removal was not different during the pandemic compared to pre-pandemic (473 ± 55 vs 500 ± 65, P = 0.19). The number of non-CDI patients who had device removal (1,851 ± 255 vs 2,135 ± 130, P = 0.01) and those with CDI not requiring surgery were significantly lower during the pandemic compared to pre-pandemic period (2,248 ± 182 vs 2,752 ± 247, P<0.001; Fig 3).

### Unadjusted outcomes for patients with CDI

Compared to others, the pandemic cohort more often had MAE (31.0 vs 28.9%, P = 0.002) while mortality rates were similar (4.8 vs 4.4%, P = 0.18). The pandemic cohort had similar time to device replacement, overall LOS, as well as post-device removal LOS, however, they had increased unadjusted hospitalization costs compared to pre-pandemic (Table 2).

**Table 2:** Unadjusted outcomes after admission for cardiac device infection during and prior to the COVID-19 pandemic.

	pandemic (n = 27,260)	pre-pandemic (n = 162,900)	P-value
Mortality (%)	4.8	4.4	0.18
Major Adverse Event (%)	31.0	28.9	0.002
Overall LOS^‡^ (days, median, IQR^†^)	7 [4–12]	7 [4–12]	0.08
Post-Device Removal LOS^‡^ (days, median, IQR^†^)	6 [3–9]	6 [4–10]	0.02
Replacement time (days, median, IQR^†^)	4 [1–7]	5 [2–7]	0.15
Hospitalization costs ($1,000s, median, IQR^†^)	20.3 [11.2–37.2]	18.5 [10.5–33.4]	<0.001

^†^IQR, *Interquartile Range*.

^‡^LOS, *Length of Stay*.

### Outcomes of patients with COVID-19 infection

Compared to those who were not infected with COVID-19 (COVID-19 negative), those who were infected (COVID-19 positive) had a higher mean Elixhauser Index score (5.8 ± 1.9 vs 5.3 ± 2.1, P = 0.01). In addition, the COVID-19 positive cohort had higher rates of diabetes (61.5 vs 49.3%, P = 0.01), and neurological disorders (24.6 vs 16.1%, P = 0.01). Both cohorts were similar in racial composition as well as hospital status (Table 3). Compared to others, COVID-19 positive patients more often had MAE (46.9 vs 30.0%, P<0.001) as well as higher mortality rates (11.5 vs 4.4%, P<0.001). They had similar time to device replacement and post device removal LOS. However, they had higher overall LOS and increased unadjusted hospitalization costs (Table 4).

**Table 3:** Baseline patient, clinical, and hospital factors after admission for cardiac device infection; (+) COVID-19, infected with COVID-19; (-) COVID-19, not infected with COVID-19.

	(-) COVID-19 (n = 189,510)	(+) COVID-19 (n = 650)	P-value
Age (years, mean ± SD^†^)	61.8 ± 15.5	63.2 ± 14.1	0.30
Female (%)	41.7	43.8	0.61
Elixhauser Index (mean ± SD^†^)	5.3 ± 2.1	5.8 ± 1.9	0.01
Device Replacement (%)	4.7	3.1	0.39
Device Removal (%)	15.6	10.0	0.09
Race (%)			0.06
White	54.1	42.3	
Black	28.2	32.3	
Hispanic	11.3	16.9	
Asian/Pacific Islander/Other	6.4	8.5	
Hospital status (%)			0.09
Urban Teaching	79.1	86.9	
Urban Non-Teaching	17.0	10.8	
Rural	3.9	2.3	
Comorbidities (%)			
Coagulopathy	15.8	20.8	0.12
Congestive Heart Failure	39.9	45.4	0.21
Diabetes	49.3	61.5	0.01
Endocarditis	11.2	13.1	0.48
Hypertension	85.3	88.5	0.34
Liver Disease	6.7	6.9	0.92
Metastatic Cancer	1.8	3.1	0.25
Neurological Disorders	16.1	24.6	0.01
Obesity	20.8	20.0	0.81
Peripheral Vascular Disease	22.3	22.3	0.9
Pulmonary Circulation Disorders	9.4	8.5	0.70
Renal Failure	66.5	74.6	0.07
Rheumatoid Arthritis	4.3	3.1	0.48
Tumor	3.4	3.8	0.80

^†^ SD, *Standard Deviation*.

**Table 4:** Unadjusted outcomes after admission for cardiac device infection; (+) COVID-19, infected with COVID-19 virus; (-) COVID-19, non-infected with COVID-19 virus.

	(-) COVID-19 (n = 189,510)	(+) COVID-19 (n = 650)	P-value
Mortality (%)	4.4	11.5	<0.001
Major Adverse Events (%)	30.0	46.9	<0.001
Overall LOS^‡^ (days, median, IQR^†^)	7 [4–12]	11 [7–21]	<0.001
Post-Device Removal LOS^‡^ (days, median, IQR^†^)	6 [3–10]	7 [5–12]	0.23
Replacement time (days, median, IQR^†^)	5 [2–7]	13 [6–14]	0.16
Hospitalization costs ($1,000s, median, IQR^†^)	18.7 [10.6–33.8]	26.9 [14.2–45.3]	<0.001

^†^IQR, *Interquartile Range*.

^‡^LOS, *Length of Stay*.

### Adjusted outcomes of patients with CDI

**Fig 4:**
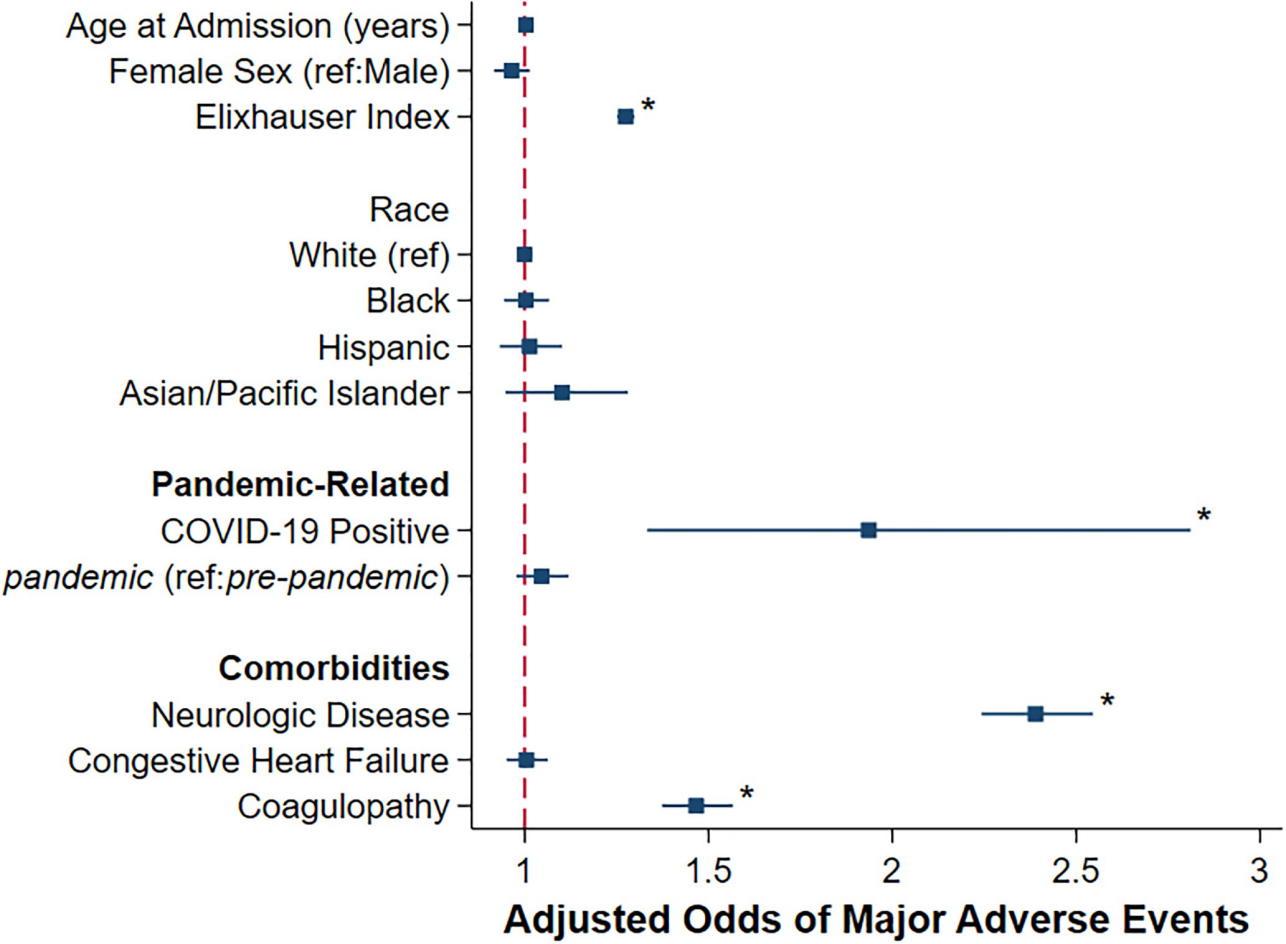
Factors associated with adjusted odds of major adverse events after admission for cardiac device infection; *P<0.*05*.

After risk adjustment, the pandemic was not associated with increased or decreased odds of MAE (AOR 1.05, 95%CI 0.98–1.12) or death (AOR 0.97, 95%CI 0.84–1.12), compared to pre-pandemic. Additionally, pandemic was linked with similar odds of device removal (AOR 0.99, 95%CI 0.90–1.10) and subsequent device replacement (AOR 0.87, 95%CI 0.74–1.03), relative to pre-pandemic. Compared to pre-pandemic, the pandemic was associated with decreased overall LOS (β -0.38 days, 95%CI -0.68, -0.08) and days to replacement (β -0.83 days, 95%CI -1.52, -0.15), but did not alter LOS following device removal (β -0.45 days, 95%CI -1.03, +0.13). Finally, the pandemic was linked with an incremental increase in adjusted hospitalization costs by $2,000 (95%CI $514–3,491). Further analysis revealed that, among other factors, infection with COVID-19 (AOR 1.94, 95%CI 1.33–2.81) and higher Elixhauser Index score (AOR 1.27, 95%CI 1.25–1.30) were associated with increased adjusted odds of MAE. However, neither congestive heart failure nor patient race was associated with increased adjusted odds of MAE (Fig 4).

## Discussion

Given the significant healthcare burden of CDI, examining access to care and clinical outcomes during the COVID-19 pandemic is particularly instructive and relevant. While prior work has characterized clinical outcomes of patients with cardiac device infection, national examination of these outcomes during the COVID-19 pandemic remains lacking [17,18]. In the present study, we found that volume of patients admitted for CDI drastically decreased during the early months of the COVID-19 pandemic and did not return to baseline. Odds of MAE in patients with CDI was similar during the pandemic compared to prior. Further, despite having a lower overall LOS, hospitalization costs were higher during the pandemic. Several of these findings warrant further discussion.

Current guidelines recommend conservative management for CDI cases that are limited to the superficial pocket or incision site [19]. However, complete device removal is imperative for cases of CDI confirmed by signs and symptoms of infection, or CDI confirmed by echocardiography and microbiological studies [20,21]. In light of the increasing volume of device placement over the past three decades, an increase in the number of CDI cases and subsequent removal could have been expected [1,22,23]. In our study, we found the overall volume of device removal to be lower during the pandemic compared to pre-pandemic. However, rates of device removal for infectious reasons remained steady following the pandemic. We also noted that volume of device removal was lower for non-CDI during the pandemic. Similarly, the volume of CDI patients who did not have device removal was lower during the pandemic compared to the preceding year. This may be explained by a lower rate of hospital presentation by patients with CDI that does not require surgery. Another plausible explanation is the lower rates of device implantation overall during the pandemic as reported by Arbelo et al [24]. The authors concluded a considerable decrease in CDI during the first wave of the pandemic between January to April 2020. Although we cannot comment on the true burden of CDI, our findings demonstrate the impact of COVID-19 pandemic related strains of medical care on the care of patients with CDI. Additionally, a study by Lechner et al reported worst myocardial damage in patients with STEMI who presented during the COVID-19 pandemic compared to pre-pandemic [25]. They attributed these poor outcomes to the COVID-19 pandemic restrictions, likely influencing delayed presentation. Further studies to evaluate longitudinal outcomes in patients with delayed diagnosis and treatment of CDI are warranted.

In the present study we found that the time from admission to device removal for patients with CDI was 0.83 days earlier during the pandemic compared to the years prior. However, despite earlier device removal, the odds of MAE remained similar. This finding may differ from previous studies that have shown early device removal to be associated with lower mortality. In a study of 416 patients, Le and colleagues reported a 3 times higher mortality risk in CDI patients who had a 12-day delay in device removal [26]. The time to device removal during the pandemic was shorter, albeit to a minor degree, possibly due to efforts to expedite discharge and avoid overcrowding. Expectedly, in the present work we noted higher odds of mortality in COVID-19 positive patients compared to their counterparts. Our results are consistent with prior work that associates COVID-19 infection with greater mortality in patients with cardiovascular disease [27]. Further investigation into outcomes of patients with CDI who are concurrently infected with COVID-19 is warranted to examine adverse events in the long term.

In the present work we noted a $2,000 incremental increase in hospitalization costs during the pandemic compared to the period prior. This may be explained by a higher resource utilization such as personal protective equipment and other measures to prevent viral transmission. Furthermore, we observed a 0.38-day decrement in overall length of stay during the pandemic compared to prior years. Space limitations at medical facilities across the US during the pandemic, may have contributed to hastened discharges. Our study highlights the higher cost burden that the pandemic imposed on the healthcare system despite shorter length of stay. Pandemic conditions aside, future work should examine the economic impact of cardiac device infections on the healthcare system and seek strategies to mitigate costs of care.

## Study limitations

This study has several limitations that must be acknowledged. As a retrospective study, we cannot establish any causal relationships. Due to the methodological limitations, this study was not able to identify the time interval between initial infection of the cardiac device and hospitalization for cardiac device infection. The ICD-10 diagnosis code that was selected is for any type of cardiac device infection (T82.7XXA) and does not distinguish the type of cardiac device that was infected. Additionally, the NIS database does not have certain granular data such as initial time to presentation, lab values, or specific organisms that limit our assessment of infection severity. In addition, we are unable to assess when patients had their initial device placed. We addressed these limitations by application of validated and rigorous statistical methods to mitigate their effects on our analyses.

## Conclusion

In summary, the pandemic was not associated with increased or decreased adjusted odds of cardiac device removal rates for CDI. Among those who had a device removal during the pandemic, time from admission to procedure was shorter. Despite higher hospitalization costs, the pandemic did not have a significant impact on clinical outcomes in patients that were admitted for CDI.

## Supporting information

S1 Checklist STROBE statement—Checklist of items that should be included in reports of observational studies. (DOCX)
